# Wrinkled Graphene–AgNWs Hybrid Electrodes for Smart Window

**DOI:** 10.3390/mi8020043

**Published:** 2017-02-01

**Authors:** Ki-Woo Jun, Jong-Nam Kim, Jin-Young Jung, Il-Kwon Oh

**Affiliations:** Creative Research Initiative Centre for Functionally Antagonistic Nano-Engineering, Department of Mechanical Engineering, Korea Advanced Institute of Science and Technology (KAIST), 291 Daehak-ro, Yuseong-gu, Daejeon 34141, Korea; jkw82@kaist.ac.kr (K.-W.J.); jnkim4388@kaist.ac.kr (J.-N.K.); jinyjung@kaist.ac.kr (J.-Y.J.)

**Keywords:** graphene, silver-nanowires, wrinkles, elastomer actuators, tunable transparency

## Abstract

Over the past few years, there has been an increasing demand for stretchable electrodes for flexible and soft electronic devices. An electrode in such devices requires special functionalities to be twisted, bent, stretched, and deformed into variable shapes and also will need to have the capacity to be restored to the original state. In this study, we report uni- or bi-axially wrinkled graphene–silver nanowire hybrid electrodes comprised of chemical vapor deposition (CVD)-grown graphene and silver nanowires. A CVD-grown graphene on a Cu-foil was transferred onto a bi-axially pre-strained elastomer substrate and silver nanowires were sprayed on the transferred graphene surface. The pre-strained film was relaxed uni-(or bi-)axially to produce a wrinkled structure. The bi-axially wrinkled graphene and silver nanowires hybrid electrodes were very suitable for high actuating performance of electro-active dielectric elastomers compared with the wrinkle-free case. Present results show that the optical transparency of the highly stretchable electrode can be successfully tuned by modulating input voltages.

## 1. Introduction

Smart windows, which can control the level of light transmission, have recently been attracting great interest, because they can be applied to a variety of applications, including vehicle windows, exterior wall windows and skylight windows. Until now, most smart window technology has been based on the redox reaction of a molecular element and a chromic material in response to external stimuli such as light, electricity, or temperature [1,2,3,4]. However, such materials are chemically unstable during the conversion processes, and are further disadvantageous in that they are difficult to control [4]. As a result, there is a need for a new smart window that is efficient in manufacturing, simple to implement, durable to operate and has a fast response time.

Among various approaches to smart window technology, there has been recent interest in techniques for modulating transmittance by changing the surface morphology of an elastomer actuator. In order to generate such a morphological change of soft materials, a flexible and stretchable conducting material should be used as compliant electrodes, which can be stretched or shrunk according to the movement of the soft material, such as nanowires, graphene, and hybrid materials [5,6,7,8,9,10,11,12]. In particular, techniques using a soft dielectric elastomer and stretchable electrodes are being studied for controlling surface morphology by applying electric input voltages. Smart window technology based on such soft materials can be applied to micro-lens arrays [13,14], flexible electronic devices [15,16], and variable diffraction gratings [17].

However, since most devices were operated with flat electrodes, the changes in transmittance were not so large, resulting in a limited application. In order to resolve this performance problem, research on smart windows using graphene and silver nanowire hybrid electrodes has been carried out, but a wrinkled structure implemented with stretchable hybrid materials has not been reported yet.

In this study, we developed a wrinkled hybrid electrode, comprised of CVD-grown graphene and silver nanowires, and reported a tunable transmittance by applying electrical fields to the dielectric elastomer actuators sandwiched with the wrinkled graphene–silver nanowire hybrid electrodes. A monolayer of graphene grown by CVD on a Cu-foil was transferred onto a bi-axially pre-strained elastomer substrate and silver nanowires were sprayed on the transferred graphene surface. The pre-strained film was released bi-(or uni-)axially to develop a wrinkled structure. The morphological and electrical characteristics of the wrinkled structures with bi-(or uni-)axially compressive strains were investigated using scanning electron microscopy (SEM). Also, the bi-axially wrinkled graphene and silver nanowire hybrid nanostructures were successfully applied to produce high actuating performance compared with the wrinkle-free case. Using the fabricated electrode, we demonstrated a device whose optical transparency was tunable with input voltages.

## 2. Preparation and Experiment

### 2.1. Materials

VHB 4905 film (3M Corp., Maplewood, MN, USA) is an appropriate material for dielectric elastomer actuators because the film is easy to handle, and both sides of the film are sticky enough for coating with electrode materials such as graphene and silver nanowires. In addition, the VHB 4905 is almost transparent, with a thickness of around 5 mm and a density of 960 kg/m^3^. The dielectric constant of the VHB 4905 film is 3.21 at 1 kHz. The dissipation factor and dielectric breakdown strength are 0.0214 and 630 V/mm, respectively [18,19].

Silver nanowires (AgNWs) are nanostructures, generally with a diameter of ten to several hundred nanometers and lengths of tens of microns. Silver nanowire material is a kind of grayish powder and is often dispersed in solvents, such as water, ethanol, and isopropanol [20]. The silver nanowires used in this study were synthesized by the polyol process [21,22,23]. A cleaned two-neck flask was placed in a heating mantle. Polyvinylpyrrolidone (PVP, Mw = 55,000) 5.86 g in 190 mL of glycerol was heated at 150 °C for 2 h to remove moisture in the PVP. The solution was injected into the cleaned two-neck flask and cooled down to 55 °C. A mixture of 0.059 g of sodium chloride, 0.5 mL of deionized water, and 10 mL of glycerol was added into the prepared solution with 1.58 g silver nitrate. The suspension was heated up to 155 °C and left for 20 min for the additional reaction. The final product was washed using glass filters and stored in methanol.

### 2.2. Fabrication

**Figure 1:**
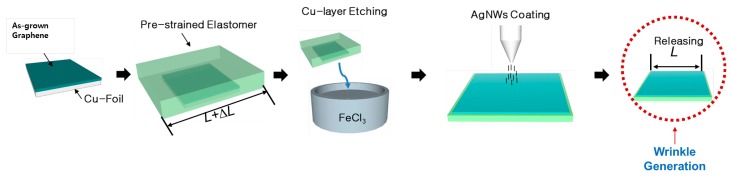
Fabrication process of the wrinkled graphene–AgNWs hybrid electrode.

The fabrication process of the graphene and silver nanowire hybrid electrode with a wrinkled structure is illustrated in Figure 1. In this process, the wrinkled structure of the elastomer surface was formed mainly due to the difference in modulus between the elastomer film and the graphene and silver nanowire electrode.

The first step is the transfer of the CVD-grown graphene onto the surface of the pre-stretched elastomer film. In this step, the copper layer is removed using a wet-etching process to allow the graphene transfer. In order to maintain electrical conductivity between the graphene islands on the elastomer surface, silver nanowires were sprayed on the wrinkled graphene surface. Then, the pre-stretched elastomer film was released, to prepare a hybrid electrode with a wrinkled structure. In order to fabricate a dielectric elastomer actuator with a wrinkled structure, the electrode formation method described above was also applied to the opposite side of the elastomer. Using this method, a wrinkled elastomer actuator whose light transmittance could be controlled was manufactured.

### 2.3. Experiment Method

To induce the electro-mechanical deformation of the dielectric elastomer actuator, a high input voltage over 1 kV needs to be applied to both electrodes. For this purpose, a high voltage power supply (Trek Model 610E, Lockport, NY, USA) was connected to both the top and bottom electrodes of the actuator. To connect each compliant electrode to a cable from the power supply, conductive copper tape was used instead of the conventional cable-wires. Actuation testing was performed on an experiment table, and video data was acquired using a complementary metal-oxide-semiconductor (CMOS) image sensor to show the areal expansion of the actuator. To observe the variation in optical transmittance under actuating conditions, we placed the fabricated actuator on the sample holder of a UV-VIS spectroscopy. The optical transmittance was then examined, as input voltages were applied to the actuator.

## 3. Results and Discussions

### 3.1. Uni- and Bi-axially Wrinkled Electrode

**Figure 2:**
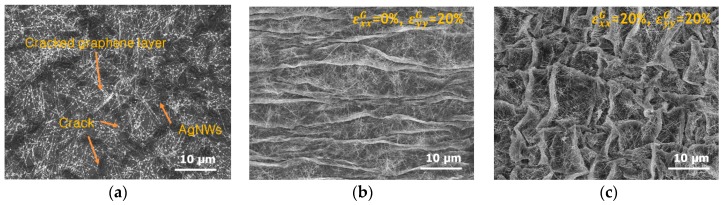
SEM images of the graphene–AgNWs hybrid electrode: (**a**) flat surface morphology, (**b**) uniaxially compressive strain of 20% and (**c**) biaxially compressive strain of 20%.

Figure 2 shows SEM images of the hybrid electrode, which forms a wrinkled structure in the uni- and bi-axial directions on the elastomer film. Because the as-transferred graphene layer is fragmented, it has low electrical conductivity, and so by itself cannot function as an actuator electrode. In order to solve this problem, we additionally used silver nanowires to act as conductive bridges between the graphene islands. Silver nanowires with a length of about 10 to 20 µm were used, and the synthesized silver nanowires were thermally annealed at a temperature of 120 °C. 

In Figure 2b,c, the surface morphology of the wrinkled structure formed along the uni- and bi-directions can be observed. A stretching–releasing method was used to create the wrinkled structure of the hybrid electrode. Pre-stretching was used to elongate the state of the elastomer film, which was used as the elastic body of the actuator. Different wrinkled structures can be formed depending on the pre-stretching direction. In this experiment, we formed wrinkled structures with uni- and bi-directions. The wavelength of the wrinkles is about 5 to 15 µm, as shown in Figure 2b,c.

### 3.2. Morphology Variation with Tensile Strength

**Figure 3:**
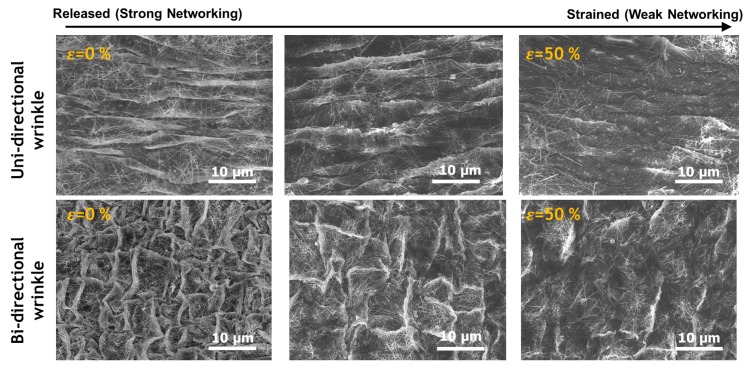
Morphology changes in the electrode surfaces with mechanical tensile strains.

The working principle of the elastomer actuator is based on an attraction force which is generated between the two electrodes. This force causes an expansion of the electrode area in the in-plane direction. In order to examine the change of the surface in the multilayered elastomer-electrodes according to the pulling forces in the in-plane direction, the surface morphology was examined under tensile conditions, as shown in Figure 3. The uni- or bi-axial mechanical forces were applied to make uni- or bi-axially wrinkled patterns. The upper figure shows a wrinkled structure formed in one axis direction, and the lower figure shows a wrinkle structure formed in two axis directions. The surface patterns were observed as 0 to 50% tensile strain applied along the wrinkle direction. As the strain value increases, it can be seen that the wrinkles spread out. In the strained condition, it was confirmed that the interval between the pitches is increased, the height of the wrinkles and the density of electrical networks are lowered.

### 3.3. Electrical Measurement

**Figure 4:**
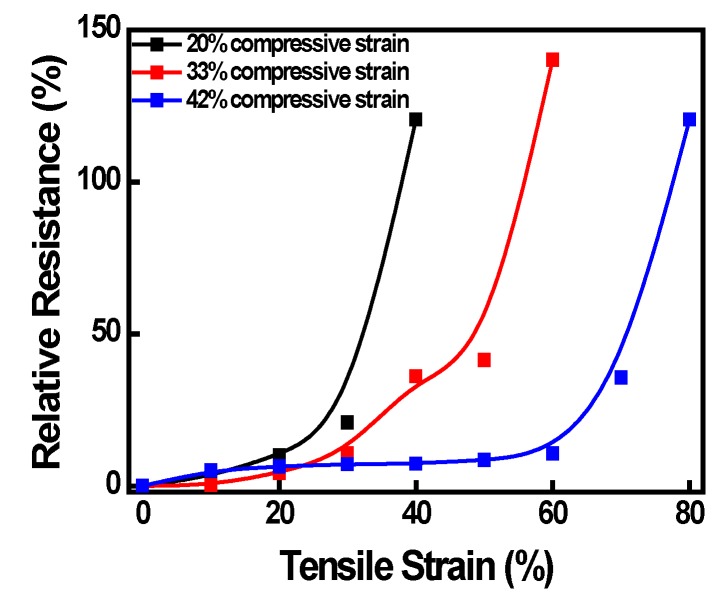
Resistance changes of wrinkled hybrid electrodes according to mechanical tensile strains.

The relative resistance of the electrodes was examined in order to investigate the influence of the wrinkling morphology. Three types of specimens with different compressive strain values were prepared for this experiment. The compressive strain values applied to the specimens were 20%, 33% and 42%, respectively. As shown in Figure 4, the relative resistance (Δ*R*/*R*_0_) remains stable in the initial section, and as the strain value is increased, the magnitude of the relative resistance value is changed sharply. These results can be explained based on the wrinkled structure of the electrode. As shown in Figure 3, when the value of the tensile strain is increased, the hybrid electrode material, that is, the connectivity between the graphene and the silver nanowires, is weakened. For this reason, it can be understood that the resistance increases sharply in the section where there are fewer wrinkled structures.

### 3.4. Electromechanical Transducer with Elastomer Films

**Figure 5:**
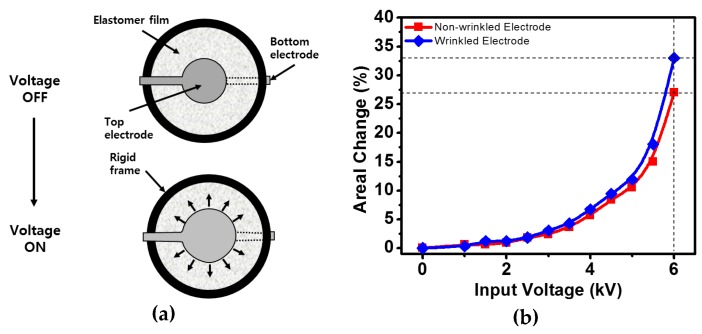
Actuation behaviors: (**a**) concept of isotropic elastomer actuation, (**b**) comparison of areal changes of wrinkle-free and wrinkled elastomer actuator.

The dielectric elastomer actuator has a sandwich structure consisting of an elastomer film with two electrodes on both sides. For application, the elastomer film was fixed in a rigid frame made of polyacetal. Figure 5a illustrates the working principle of the elastomer actuator. When an input voltage is applied to the electrodes, electrons are accumulated on both sides of the wrinkled electrode. At this time, Maxwell stress is generated in the thickness direction of the elastomer film. Deformation occurs in the in-plane direction from the active area where the electrodes are formed.

In order to verify the operating performance of the actuator, we connected the electrode of the actuator to a DC high voltage supply capable of generating 0–10 kV. Then, the applied voltage was gradually increased from 0 to 6 kV, and the change in the area of the actuator was examined. For more precise performance analysis, it is essential to determine the area of the increased actuator under electrical stimuli. For this purpose, the moving images of the actuator were captured frame-by-frame, and analyzed using an image processing program. The actuation of conventional dielectric elastomer actuators using a high input-voltage caused the electrodes to expand with an isotropic areal strain as shown in Figure 5a. Isotropic behaviors of the dielectric elastomer actuator can be differently realized by the morphological configuration of hybrid electrodes. Figure 5b shows the experimental results to confirm the performance difference of the wrinkled and wrinkle-free electrodes when electrical input voltages are applied to two dielectric elastomer actuators. The actuator sample with wrinkled electrodes shows an areal change (Δ*A*/*A*_0_) of 33%. Within 4 kV, the areal change of the actuator is almost similar in both cases, but there is a remarkable maximum difference of about 6% at 6 kV.

**Figure 6:**
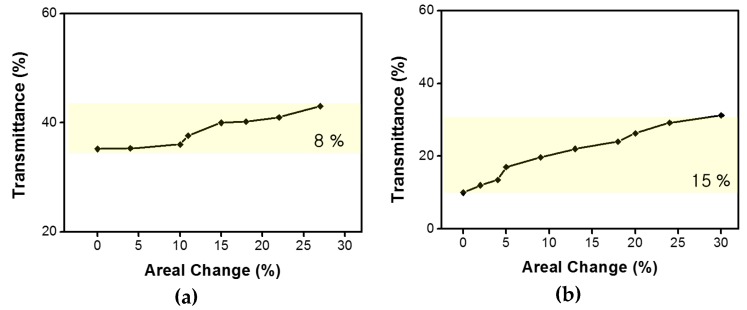
Optical transmittance control of (**a**) a wrinkle-free and (**b**) wrinkled elastomer actuator with electrical input.

As the applied voltage increases, the strength of the interaction force in the thickness direction increases in the elastomer actuator. This force has the effect of reducing the thickness of the film and increasing the area of the electrode. At this time, the transmittance of the active area is increased due to the reduced thickness of the film and the change in surface morphology on the surface of the elastomer film. Figure 6 is a graphical representation of the relationship between the rate of areal change and transmittance obtained when a voltage is applied to the actuator. Using a sample without the wrinkled electrodes, experimental results confirmed that the areal change rate was about 27% and the transmittance at 550 nm was changed by about 8%.

Using the same method as that used for the wrinkle-free sample, the change in active area of the dielectric elastomer actuator with wrinkled graphene–AgNWs hybrid electrodes was measured with respect to the applied voltages. Then, the transmittance change according to area changes was also examined. The wrinkled electrode structure exhibited about a 33% change in area ratio, and a transmittance change of about 15%. That is, although the wrinkled structure showed the same area change rate, it can be confirmed that the transmittance was about twice as large as that in the case of the wrinkle-free sample. When compared with the structure without wrinkles, the cause of this phenomenon is understood to be the difference in transmittance due to the scattering of light.

## 4. Conclusions

A novel dielectric elastomer actuator with wrinkled graphene and silver nanowire hybrid electrodes was developed in this study. Graphene and silver nanowires were used to realize the highly conductive and compliant electrodes. The developed dielectric elastomer actuator with the wrinkled surfaces exhibited a large in-plane deformation of up to 33%. As compared with wrinkle-free electrodes, the proposed actuator with wrinkled electrodes exhibited a 10% higher areal change rate because of the synergistic effect of the ultrahigh conductivity of the silver nanowires and the remaining capacitance of the graphene under large deformation. This novel elastomer actuator with wrinkled hybrid electrodes may be a candidate to tune the transmittance of a smart window system.

## References

[1] Beaujuge P.M., Reynolds J.R. (2010). Color control in π-conjugated organic polymers for use in electrochromic devices. Chem. Rev..

[2] Bechinger C., Ferrer S., Zaban A., Sprague J., Gregg B.A. (1996). Photoelectrochromic windows and displays. Nature.

[3] Granqvist C.G. (2000). Electrochromic tungsten oxide films: Review of progress 1993–1998. Sol. Energy Mater. Sol. Cells.

[4] Lampert C.M. (2004). Chromogenic smart materials. Mater. Today.

[5] An B.W., Gwak E.-J., Kim K., Kim Y.-C., Jang J., Kim J.-Y., Park J.-U. (2016). Stretchable, transparent electrodes as wearable heaters using nanotrough networks of metallic glasses with superior mechanical properties and thermal stability. Nano Lett..

[6] An B.W., Hyun B.G., Kim S.-Y., Kim M., Lee M.-S., Lee K., Koo J.B., Chu H.Y., Bae B.-S., Park J.-U. (2014). Stretchable and transparent electrodes using hybrid structures of graphene–metal nanotrough networks with high performances and ultimate uniformity. Nano Lett..

[7] He T., Xie A., Reneker D.H., Zhu Y. (2014). A tough and high-performance transparent electrode from a scalable and transfer-free method. ACS Nano.

[8] Hsu P.-C., Wang S., Wu H., Narasimhan V.K., Kong D., Ryoung Lee H., Cui Y. (2013). Performance enhancement of metal nanowire transparent conducting electrodes by mesoscale metal wires. Nat. Commun..

[9] Hsu P.-C., Wu H., Carney T.J., McDowell M.T., Yang Y., Garnett E.C., Li M., Hu L., Cui Y. (2012). Passivation coating on electrospun copper nanofibers for stable transparent electrodes. ACS Nano.

[10] Kim J., Lee M.-S., Jeon S., Kim M., Kim S., Kim K., Bien F., Hong S.Y., Park J.-U. (2015). Highly transparent and stretchable field-effect transistor sensors using graphene–nanowire hybrid nanostructures. Adv. Mater..

[11] Kim K., Kim J., Hyun B.G., Ji S., Kim S.-Y., Kim S., An B.W., Park J.-U. (2015). Stretchable and transparent electrodes based on in-plane structures. Nanoscale.

[12] Wu H., Kong D., Ruan Z., Hsu P.-C., Wang S., Yu Z., Carney T.J., Hu L., Fan S., Cui Y. (2013). A transparent electrode based on a metal nanotrough network. Nat. Nanotechnol..

[13] Chan E.P., Crosby A.J. (2006). Fabricating microlens arrays by surface wrinkling. Adv. Mater..

[14] Chandra D., Yang S., Lin P.-C. (2007). Strain responsive concave and convex microlens arrays. Appl. Phys. Lett..

[15] Khang D.-Y., Jiang H., Huang Y., Rogers J.A. (2006). A stretchable form of single-crystal silicon for high-performance electronics on rubber substrates. Science.

[16] Scalisi R.G., Paleari M., Favetto A., Stoppa M., Ariano P., Pandolfi P., Chiolerio A. (2015). Inkjet printed flexible electrodes for surface electromyography. Org. Electron..

[17] Harrison C., Stafford C.M., Zhang W., Karim A. (2004). Sinusoidal phase grating created by a tunably buckled surface. Appl. Phys. Lett..

[18] Opris D.M., Molberg M., Walder C., Ko Y.S., Fischer B., Nüesch F.A. (2011). New silicone composites for dielectric elastomer actuator applications in competition with acrylic foil. Adv. Funct. Mater..

[19] Jun K.W., Lee J.M., Lee J.Y., Oh I.K. (2014). Bio-inspired dielectric elastomer actuator with agnws coated on carbon black electrode. J. Nanosci. Nanotechnol..

[20] Tien H.-W., Hsiao S.-T., Liao W.-H., Yu Y.-H., Lin F.-C., Wang Y.-S., Li S.-M., Ma C.-C.M. (2013). Using self-assembly to prepare a graphene-silver nanowire hybrid film that is transparent and electrically conductive. Carbon.

[21] Jin J., Lee J., Jeong S., Yang S., Ko J.-H., Im H.-G., Baek S.-W., Lee J.-Y., Bae B.-S. (2013). High-performance hybrid plastic films: A robust electrode platform for thin-film optoelectronics. Energy Environ. Sci..

[22] Lee J., Lee I., Kim T.-S., Lee J.-Y. (2013). Efficient welding of silver nanowire networks without post-processing. Small.

[23] Yang C., Gu H., Lin W., Yuen M.M., Wong C.P., Xiong M., Gao B. (2011). Silver nanowires: From scalable synthesis to recyclable foldable electronics. Adv. Mater..

